# Prognostic value of inflammatory markers and clinical features for survival in advanced or metastatic esophageal squamous cell carcinoma patients receiving anti-programmed death 1 treatment

**DOI:** 10.3389/fonc.2023.1144875

**Published:** 2023-03-23

**Authors:** Liangshan Da, Ziting Qu, Congjun Zhang, Yuanyuan Shen, Wei Huang, Yiyin Zhang, Kangsheng Gu

**Affiliations:** Department of Oncology, The First Affiliated Hospital of Anhui Medical University, Hefei, China

**Keywords:** esophageal squamous cell carcinoma, PD-1, inflammatory markers, prognosis, immunotherapy

## Abstract

**Purpose:**

This study aims to assess the prognostic value of inflammatory markers and clinical features in advanced or metastatic esophageal squamous cell carcinoma (ESCC) patients receiving anti-programmed death 1 (PD-1) treatment.

**Methods:**

Based on receiver operating characteristic curve (ROC) analysis, Youden’s indexes were applied to determine the cut-off values for inflammatory markers, including neutrophil-to-lymphocyte ratio (NLR), derived neutrophil-to-lymphocye ratio (dNLR), monocyte-to-lymphocyte ratio (MLR), platelet-to-lymphocyte ratio (PLR), and systemic immune-inflammation index (SII). Wilcoxon test was conducted to evaluate the changes in above inflammatory markers. Kaplan-Meier method was utilized to estimate progression-free survival (PFS) and overall survival (OS), and the Log-rank test was used to compare the different survival between groups. Univariate and multivariate Cox regression analyses were performed to assess the prognostic value of inflammatory markers and clinical features.

**Results:**

162 advanced or metastatic ESCC patients receiving anti-PD-1 treatment were enrolled in this retrospective study. The cut-off values of NLR, dNLR, MLR, PLR, and SII were 4.748, 2.214, 0.309, 250.505, and 887.895, respectively. NLR, dNLR, PLR, and SII declined significantly among the partial response (PR) (P<0.001, P<0.001, P=0.036, P<0.001), objective response rate (ORR) (P<0.001, P<0.001, P=0.036, P<0.001), and disease control rate (DCR) (P<0.001, P<0.001, P=0.038, P<0.001) groups, respectively. Significant increases were found in NLR (P<0.001), dNLR (P<0.001), MLR (P=0.001), and SII (P=0.024) when anti-PD-1 treatment failed. Multivariate Cox regression analysis indicated that NLR (P<0.001, P=0.002), lymph node metastasis (P=0.013, P=0.001), Eastern Cooperative Oncology Group Performance Status (ECOG PS) (P=0.008, P=0.002), and treatment lines (P=0.037, P=0.048) were significant prognostic indicators of PFS and OS. Additionally, SII (P=0.016) was also significantly related to OS in ESCC patients. The risk score model showed that low risk patients prolonged PFS and OS than those with middle or high risk (P<0.001, P<0.001).

**Conclusion:**

Inflammatory markers can reflect short-term outcomes of anti-PD-1 treatment for ESCC patients. NLR, lymph node metastases, ECOG PS, and treatment lines are significant prognostic indicators for PFS and OS. And the risk score model constructed based on the above factors has favourable prognostic predictive value.

## Introduction

Esophageal cancer (EC) is one of the highly prevalent and aggressive malignancies worldwide, which gravely threatens the health of humans (1). Approximately 90% of EC cases in Asian populations are ESCC (2). Nearly 50% of global ESSC cases occur in China (3). Because of lacking early specific symptoms, numerous patients are confirmed as the advanced or metastatic stage at diagnosis and lose the opportunity for surgery (4). However, the effectiveness of current chemotherapy and other treatments for patients with advanced ESCC is not satisfactory. The prognosis of these patients is poor, with an overall 5-year survival rate of less than 15% (5).

In recent years, anti-PD-1 treatment has made a great breakthrough in the treatment of ESCC and has changed the treatment strategy dramatically. Numerous phase III trials have demonstrated the survival benefits of immunotherapy in ESCC (6). In the first-line therapy, studies on ESCORT-1st (7), ORIENT-15 (8), and JUPITER-06 (9) demonstrated that immunotherapy together with chemotherapy can significantly prolong PFS and OS in patients with advanced or metastatic ESCC. In the second-line treatment, the results of KEYNOTE-181 (10), ESCORT (11), and RATIONALE-302 (12) showed a benefit of immunotherapy compared to chemotherapy in terms of OS. Some patients, however, could not benefit from the treatment because of considerable heterogeneity in tumor tissue and immunity. Currently, the common biomarkers for immunotherapy are programmed cell death ligand-1 (PD-L1) expression, tumor mutational burden (TMB), and microsatellite instability (MSI) status (13, 14). Unfortunately, these biomarkers depend on tumor tissue and molecular analyses, and these analyses are complex and expensive, so they have limited predictive value in clinical practice. Developing non-invasive and inexpensive biomarkers to screen patients who can benefit from immunotherapy is urgently required.

It is well known that inflammation is one of the hallmarks of cancer, which contributes to tumorigenesis, progression, as well as metastasis (15–17). The number of neutrophils, lymphocytes, and platelets in the circulating blood can reflect the body’s immune inflammatory state (18). There are increasing evidence that systemic inflammatory biomarkers can serve as prognostic indicators in various cancers (19–21). Meta-analyses have shown that the inflammatory biomarkers have similar prognostic value in non-small cell lung cancer (NSCLC) (22, 23), renal cancer (24), as well as melanoma (25) patients treated with immunotherapy. Nevertheless, there are limited studies on the relationship between prognosis and inflammatory biomarkers in ESCC patients receiving immunotherapy. Additionally, it is noteworthy that the level of inflammatory biomarkers may be altered by treatment. However, most studies have primarily focused on their baseline levels rather than on their dynamic changes. Most important of all, until now, no prognostic scoring system that can provide multiple information including inflammation, immunity and clinical features has been established. Therefore, our study aims to comprehensively assess the relationship between the changes in NLR, dNLR, MLR, PLR, and SII and the short-term outcomes of advanced or metastatic ESCC patients receiving anti-PD-1 treatment, and to further explore the prognostic value of the aforementioned inflammatory biomarkers and clinical features of patients.

## Materials and methods

### Patients selection and data collection

Patients with advanced or metastatic ESCC receiving anti-PD-1 treatment at the First Affiliated Hospital of Anhui Medical University between August 20, 2019 and February 28, 2022 were selected in our study. Ethics Committee approval (NO. Quick-PJ 2022-14-35) was obtained by our hospital for this study.

Here are the criteria of inclusion (1): histologically or cytologically confirmed ESCC; (2) patients with advanced or metastatic cancer (patients had unresectable, or recurrent disease that precluded esophagectomy or curative chemoradiotherapy or radical radiotherapy, or distant metastatic disease); (3) patients who received anti-PD-1 treatment; (4) sufficient clinical data.

Here are the criteria of exclusion: (1) patients with other cancers or other types of pathology; (2) patients enrolled in clinical trials (Because the treatment regimen has not yet been unblinded, we cannot determine whether patients received placebo or immunotherapy); (3) patients had no blood examination results at baseline; (4) patients had no medical images for estimating effectiveness; (5) patients with acute or severe autoimmune disease, or blood disease.

**Figure 1:**
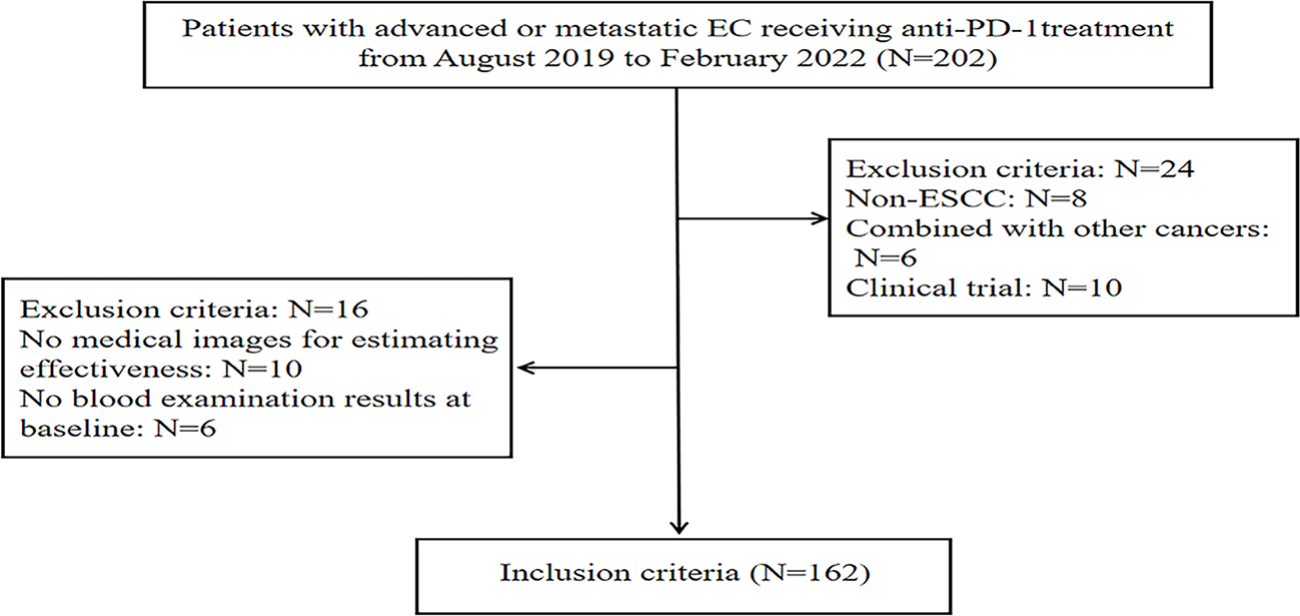
The flowchart of the patient selection process.

Ultimately, 40 patients were excluded from this study and 162 patients were enrolled. The flow chart of patient selection was revealed in **Figure 1**. Two investigators independently conducted data extraction, including age, gender, tumor locations, metastatic sites, ECOG PS, history of drinking and smoking, prior operation, treatment lines, PD-1 inhibitors, and treatment type. The tumor-node-metastasis (TNM) stage was evaluated according to the AJCC TNM staging system (the 8th edition). All data were provided primarily by an electrical clinical medical record system.

### Definition of the inflammatory markers

Patients’ peripheral blood samples were dynamically collected at three periods: baseline, optimal effect, and when the disease progressed. The test results of white blood cell (WBC), neutrophil (NE), lymphocyte (LY), monocyte (MO), and platelet (PLT) in peripheral blood were recorded to compute the values of inflammatory markers. The definitions of NLR, dNLR, MLR, PLR, and SII were described as follows: NLR = NE/LY; dNLR = NE/(WBC-NE); MLR = MO/LY; PLR = PLT/LY; SII = NE*PLT/LY. Based on ROC analysis, the cut-off values were determined for inflammation markers using Youden’s index.

### Evaluation of efficacy

We performed computed tomography scans or other examinations to assess treatment response every 2 cycles or when patients developed severe clinical symptoms. As defined by Response Evaluation Criteria in Solid Tumors version 1.1 (RECIST 1.1) or iRECIST, short-term outcomes were classified as complete response (CR), partial response (PR), stable disease (SD), and progressive disease (PD). In order to exclude pseudo-progression, we reconfirmed the evaluation of PD patients according to iRECIST. Two authors independently extracted efficacy evaluation information from the electronic medical records, which was further verified by other doctors according to imaging information. The ORR was determined by the proportion of patients with CR and PR. DCR was determined by the proportion of patients with CR, PR, and SD. PFS was the interval time from the first day of the first cycle of anti-PD-1 treatment to disease progression or all-cause death or last follow-up. OS was computed from the first day of the first cycle of anti-PD-1 treatment until either all-cause death or the last follow-up. Patients were mainly followed up through medical record searches or telephone communications. The cut-off date was August 31, 2022.

### Statistical analyses

Inflammatory markers were categorized according to the cut-off values determined by Youden’s index using ROC analysis. Wilcoxon test was utilized to evaluate the relationship between short-time outcomes and changes in inflammatory markers. Kaplan-Meier method was applied to calculate PFS and OS of patients, and the Log-Rank test was performed to compare the different survival between groups. The univariate and multivariate Cox regression analyses were utilized for determining the prognostic indicator, and hazard ratios (HR) and 95% confidence intervals (CI) were given. The variables with P *<* 0.05 from univariate analysis were incorporated into multivariate models. P *<* 0.05 was the significance threshold, and all tests were two-sided. Statistical analyses were conducted utilizing SPSS 26.0 (SPSS Inc, Chicago, IL, USA) and pictures were drawn with R software (version 4.0.2).

## Results

### Baseline clinical features

Patients’ baseline clinical features were given in **Table 1**. Finally, 162 patients were selected in our study based on the criteria of inclusion and exclusion. The median patient age was 66 years (range: 46-85). 84% were men, 92.0% with ECOG PS of 0-1, 31.5% had a history of drinking, 36.4% had a history of smoking, and 56.8% were postoperative recurrence. The common distant metastatic sites of these patients included liver (22.2%), lymph nodes (42.0%), lung (22.8%) and bone (9.3%). The tumor locations were as follows: upper thoracic (6.2%), middle thoracic (48.8%), and lower thoracic (45.0%) respectively. All patients received combined treatment, including 59.3% with chemotherapy, 17.9% with target therapy, and 22.8% with chemotherapy and target therapy. Among all patients, 73.5% received immunotherapy at the first line, and 26.5% at the second line or posterior. PD-1 inhibitors of Camrelizumab, Sintilimab, and Toripalimab accounted for 85.8%, 11.1%, and 3.1%, respectively. The median PFS and OS were 7.8 (95% CI: 7.0-8.6) and 15.4 (95% CI: 13.0-17.7) months, respectively. The median follow-up time was 16.9 (95% CI: 14.7-19.1) months.

**Table 1:** Baseline characteristics of advanced or metastatic ESCC patients.

Characteristics	No. of patients (N =162)	Percentage (%)
Age(years)		
≤65	74	45.7
>65	88	54.3
Gender		
Male	136	84.0
Female	26	16.0
Tumor location		
Upper	10	6.2
Middle	79	48.8
Low	73	45.0
Metastatic site		
Liver metastasis		
Negative	126	77.8
Positive	36	22.2
Lymph node metastasis		
Negative	94	58.0
Positive	68	42.0
Lung metastasis		
Negative	125	77.2
Positive	37	22.8
Bone metastasis		
Negative	147	90.7
Positive	15	9.3
ECOG PS		
≤1	149	92.0
≥2	13	8.0
Drinking history		
No	111	68.5
Yes	51	31.5
Smoking history		
Never	103	63.6
Current/former	59	36.4
Prior operation		
No	70	43.2
Yes	92	56.8
Treatment lines		
1 line	119	73.5
≥2 lines	43	26.5
PD-1 inhibitors		
Camrelizumab	139	85.8
Sintilimab	18	11.1
Toripalimab	5	3.1
Treatment type		
PD-1+ Chemotherapy	96	59.3
PD-1+ Target therapy	29	17.9
PD-1+ Chemotherapy + Target therapy	37	22.8
NLR		
≤4.748	128	79.0
>4.748	34	21.0
dNLR		
≤2.214	96	59.3
>2.214	66	40.7
MLR		
≤0.309	80	49.4
>0.309	82	50.6
PLR		
≤250.505	124	76.5
>250.505	38	23.5
SII		
≤887.895	118	72.8
>887.895	44	27.2

### The cut-off values of inflammatory markers

**Figure 2:**
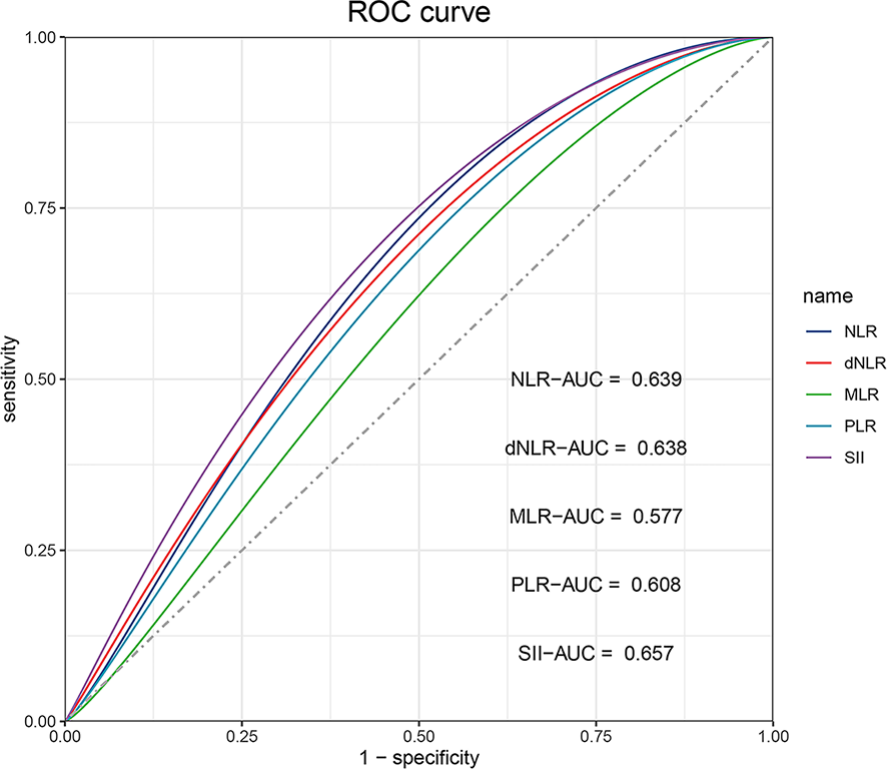
The ROC curve analysis for cut-off values of NLR, dNLR, MLR, PLR, and SII, respectively. The areas under the ROC curve of NLR, dNLR, MLR, PLR, and SII are indicated.

The NLR, dNLR, MLR, PLR, and SII of ESCC patients receiving anti-PD-1 treatment were calculated. The ROC curves were drawn according to the patient survival status, and the cut-off values of the above indicators were determined by Youden’s index according to ROC analysis. As shown in **Figure 2**, the areas under the ROC curve for NLR, dNLR, MLR, PLR, and SII were 0.639 (0.554-0.724, P=0.002), 0.638 (0.553-0.723, P=0.002), 0.577 (0.489-0.666, P=0.089), 0.608 (0.522-0.695, P=0.017), and 0.657 (0.574-0.741, P=0.001), respectively. The cut-off values of NLR, dNLR, MLR, PLR, and SII were 4.748, 2.214, 0.309, 250.505, and 887.895, respectively. According to the baseline levels of inflammatory markers, patients were classified into low (≤ cut-off values) and high groups (> cut-off values) (**Table 1**).

### Short-term outcomes for ESCC patients receiving anti-PD-1 treatment

**Figure 3:**
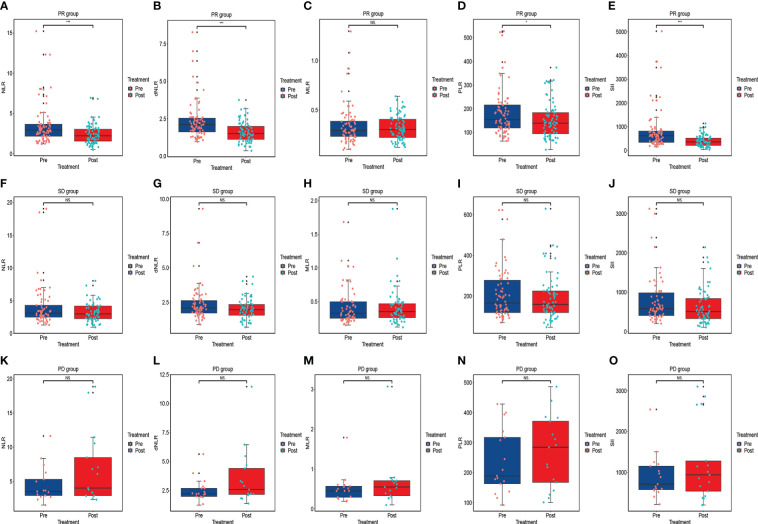
The changes in inflammatory markers in advanced or metastatic ESCC patients according to the short-term efficacy of anti-PD-1 treatment including PR group **(A–E)**, SD group **(F–J)**, and PD group **(K–O)**. NS, No statistic significance; *P < 0.05; ***P<0.001.

**Figure 4:**
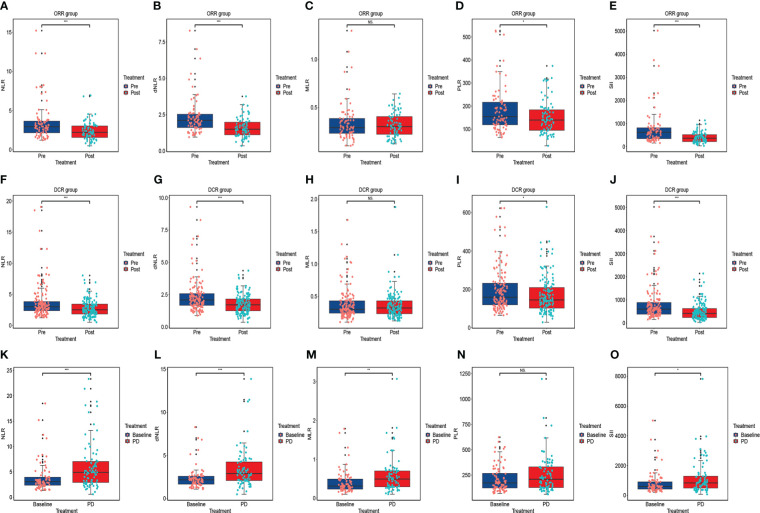
The changes in inflammatory markers in advanced or metastatic ESCC patients according to the efficacy of anti-PD-1 treatment including ORR group **(A–E)**, DCR group **(F–J)**; The changes in inflammatory markers when the failure of anti-PD-1 treatment compared to baseline **(K–O)**. ORR: the patient who obtained PR, or CR were included in this group; DCR: the patient who achieved SD, PR or CR were included in this group. NS, No statistic significance; *P < 0.05; **P < 0.01; ***P < 0.001.

Based on RECIST 1.1 or iRECIST, the number of patients with CR, PR, SD and PD were 0, 79, 66, and 17, respectively, and no patient developed pseudo-progression. The ORR and DCR were 48.8% and 89.5%. According to Wilcoxon test, NLR significantly declined among the PR (P<0.001) (**Figure 3A**), ORR (P<0.001) (**Figure 4A**), and DCR (P<0.001) (**Figure 4F**) groups. This downtrend was also found in dNLR with PR (P<0.001) (**Figure 3B**), ORR (P<0.001) (**Figure 4B**), and DCR (P<0.001) (**Figure 4G**) groups and PLR with PR (P=0.036) (**Figure 3D**), ORR (P=0.036) (**Figure 4D**), and DCR (P=0.038) (**Figure 4I**) groups. Moreover, a significant decrease of SII was also noticed in PR (P<0.001) (**Figure 3E**), ORR (P<0.001) (**Figure 4E**), and DCR (P<0.001) (**Figure 4J**) groups. However, these changes were not observed in MLR (**Figures 3C**, **4C, H**). Meanwhile, no significant changes were found in all above inflammatory markers in SD (**Figures 3F–J**) and PD (**Figures 3K–O**) groups. Up to the last follow-up date, 89 patients achieved PD due to the failure in anti-PD-1 treatment, and inflammatory markers were recorded in 82 of these patients. We further analyzed the changes in inflammatory markers in 82 PD patients. Compared to baseline, NLR (P<0.001) (**Figure 4K**), dNLR (P<0.001) (**Figure 4L**), MLR (P=0.001) (**Figure 4M**), and SII (P=0.024) (**Figure 4O**) were significantly increased when anti-PD-1 therapy failure, except for PLR (P=0.17) (**Figure 4N**).

### Evaluation of Kaplan-Meier survival curves

**Figure 5:**
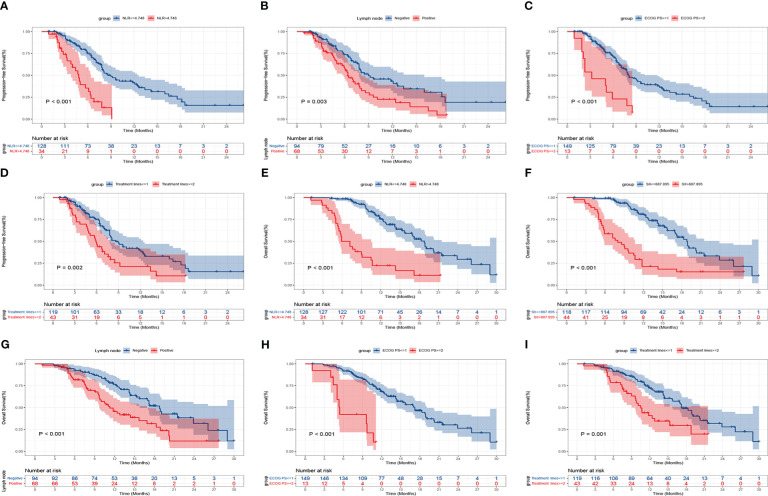
Kaplan-Meier curves of progression-free survival (PFS) and overall survival (OS) for patients with advanced or metastatic ESCC according to baseline clinical parameters. PFS: NLR **(A)**, lymph node metastasis **(B)**, ECOG PS **(C)**, treatment lines **(D)**; OS: NLR **(E)**, SII **(F)**, lymph node metastasis **(G)**, ECOG PS **(H)**, and treatment lines **(I)**.

Kaplan-Meier curves of PFS and OS were shown in **Figure 5**. In contrast to patients with a high NLR at baseline, those with a low NLR significantly improved PFS (mPFS, 8.6 months, 95% CI: 7.048-10.086, vs. 4.8 months, 95% CI: 4.042-5.625, P<0.001) (**Figure 5A**), and OS (mOS, 18.8 months, 95% CI: 16.999-20.601, vs. 5.9 months, 95% CI: 3.900-7.900, P<0.001) (**Figure 5E**). Additionally, OS was longer in patients with baseline SII ≤ 887.895 than in those with SII > 887.895 (mOS, 18.8 months, 95% CI: 16.963-20.637, vs. 8.0 months, 95% CI: 4.756-11.177, P<0.001) (**Figure 5F**). With respect to clinical features, patients with lymph node metastasis negative had a longer PFS and OS than those with lymph node metastasis positive (mPFS, 9.0 months, 95% CI: 5.514-12.486, vs. 6.4 months, 95% CI: 5.221-7.579, P=0.003; mOS, 18.8 months, 95% CI: 16.895-20.705, vs. 11.3 months, 95% CI: 9.110-13.424, P<0.001) (**Figures 5B, G**). In comparison to the low ECOG PS group, the high ECOG PS group had a worse PFS and OS (mPFS, 8.0 months, 95% CI: 6.883-9.050, vs. 3.2 months, 95% CI: 0.000-6.410, P<0.001; mOS, 17.2 months, 95% CI: 14.577-19.756, vs. 5.4 months, 95% CI: 4.772-6.095, P<0.001) (**Figures 5C, H**). Patients receiving anti-PD-1 treatment in the first-line significantly prolonged PFS and OS than those in the second-line or posterior treatment (mPFS, 8.6 months, 95% CI: 6.984-10.150, vs. 5.9 months, 95% CI: 4.684-7.116, P=0.002; mOS, 17.3 months, 95% CI: 15.369-19.298, vs. 10.5 months, 95% CI: 9.080-11.853, P=0.001) (**Figures 5D, I**).

### Prognostic value of inflammatory markers and clinical features in ESCC patients

The univariate Cox regression analysis indicated that inflammatory markers including NLR, dNLR, MLR, PLR, as well as SII at baseline and clinical features including lymph node metastasis, ECOG PS, treatment lines, and treatment type were related to PFS (P<0.05). Nevertheless, multivariate Cox analysis showed that NLR (HR: 3.095, 95% CI: 1.835-5.220, P<0.001), lymph node metastasis (HR: 1.722, 95% CI: 1.120-2.647, P=0.013), ECOG PS (HR: 2.437, 95% CI: 1.263-4.071, P=0.008), and treatment lines (HR: 1.627, 95% CI: 1.030-2.569, P=0.037) were the significant prognostic indicators for PFS (**Table 2**). Furthermore, univariate analysis showed that inflammatory markers including NLR, dNLR, MLR, PLR, as well as SII at baseline and clinical features including lymph node metastasis, ECOG PS, and treatment lines were linked to OS (P<0.05). However, after multivariate analysis, the result indicated that NLR (HR: 2.736, 95% CI: 1.451-5.159, P=0.002), SII (HR: 2.068, 95% CI: 1.144-3.739, P=0.016), lymph node metastasis (HR: 2.153, 95% CI: 1.364-3.398, P=0.001), ECOG PS (HR: 3.454, 95% CI: 1.600-7.457, P=0.002), and treatment lines (HR: 1.665, 95% CI: 1.005-2.760, P=0.048) were the significant prognostic indicators for OS (**Table 3**).

**Table 2:** Univariate and multivariate analysis of PFS in advanced or metastatic ESCC patients receiving anti-PD-1 treatment.

	Univariate analysis		Multivariate analysis	
	HR (95% CI)	P value	HR (95% CI)	P value
Age (years)				
≤65	1 (reference)			
>65	0.863 (0.566-1.316)	0.492	–	–
Gender				
Male	1 (reference)			
Female	1.017 (0.582-1.777)	0.954	–	–
Location		0.903		
Upper	1 (reference)			
Middle	0.730 (0.392-1.928)	0.730	–	–
Low	0.943 (0.419-2.127)	0.888	–	–
Metastatic site				
Liver metastasis				
Negative	1 (reference)			
Positive	1.525 (0.939-2.478)	0.086	–	–
Lymph node metastasis				
Negative	1 (reference)		1 (reference)	
Positive	1.874 (1.233-2.849)	0.003	1.722 (1.120-2.647)	0.013
Lung metastasis				
Negative	1 (reference)			
Positive	1.237 (0.763-2.005)	0.384	–	–
Bone metastasis				
Negative	1 (reference)			
Positive	1.039 (0.520-2.076)	0.915	–	–
ECOG PS				
≤1	1 (reference)		1 (reference)	–
≥2	3.701 (1.987-6.895)	<0.001	2.437 (1.263-4.071)	0.008
Drinking history				
No	1 (reference)			
Yes	1.072 (0.668-1.721)	0.773	–	–
Smoking history				
Never	1 (reference)		–	–
Current/former	0.991 (0.638-1.538)	0.967	–	–
Operation				
No	1 (reference)		–	–
Yes	1.330 (0.867-2.039)	0.189	–	–
Treatment lines				
1 line	1 (reference)		1 (reference)	
≥2 lines	1.995 (1.283-3.102)	0.002	1.627 (1.030-2.569)	0.037
PD-1 inhibitors		0.493		
Camrelizumab	1 (reference)		–	–
Sintilimab	0.857 (0.463-1.653)	0.681	–	–
Toripalimab	0.334 (0.046-2.432)	0.279	–	–
Treatment type		0.005	–	–
PD-1+ Chemotherapy	1 (reference)		1 (reference)	
PD-1+ Target therapy	2.320 (1.387-3.878)	0.001	1.275 (0.783-2.076)	0.329
PD-1+ Chemotherapy + Target therapy	1.348 (0.812-2.239)	0.249	–	–
NLR				
≤4.748	1 (reference)		1 (reference)	
>4.748	3.897 (2.357-6.445)	<0.001	3.095 (1.835-5.220)	<0.001
dNLR				
≤2.214	1 (reference)		1 (reference)	
>2.214	1.873 (1.228-2.856)	0.003	1.176 (0.707-1.955)	0.533
MLR				
≤0.309	1 (reference)		1 (reference)	
>0.309	1.574 (1.033-2.398)	0.033	0.883 (0.511-1.525)	0.655
PLR				
≤250.505	1 (reference)		1 (reference)	
>250.505	1.632 (1.026-2.596)	0.037	0.843 (0.492-1.445)	0.535
SII				
≤887.895	1 (reference)		1 (reference)	
>887.895	2.165 (1.390-3.373)	<0.001	1.088 (0.523-2.264)	0.821

**Table 3:** Univariate and multivariate analysis of OS in advanced or metastatic ESCC patients receiving anti-PD-1 treatment.

	Univariate analysis		Multivariate analysis	
	HR (95% CI)	P-value	HR (95% CI)	P-value
Age (years)				
≤65	1 (reference)			
>65	0.888 (0.574-1.374)	0.593	–	–
Gender				
Male	1 (reference)			
Female	0.689 (0.372-1.279)	0.235	–	–
Location		0.846		
Upper	1 (reference)			
Middle	1.313 (0.515-3.347)	0.326	–	–
Low	1.295 (0.505-3.321)	0.591	–	–
Metastatic site				
Liver metastasis				
Negative	1 (reference)			
Positive	1.484 (0.902-2.441)	0.118	–	–
Lymph node metastasis				
Negative	1 (reference)		1 (reference)	
Positive	2.163 (1.393-3.357)	<0.001	2.153 (1.364-3.398)	0.001
Lung metastasis				
Negative	1 (reference)			
Positive	1.360 (0.847-2.182)	0.201	–	–
Bone metastasis				
Negative	1 (reference)			
Positive	1.058 (0.528-2.120)	0.873	–	–
ECOG PS				
≤1	1 (reference)		1 (reference)	–
≥2	6.529 (3.206-13.299)	<0.001	3.454 (1.600-7.457)	0.002
Drinking history				
No	1 (reference)		–	–
Yes	0.990 (0.603-1.627)	0.969	–	–
Smoking history				
Never	1 (reference)			
Current/former	1.049 (0.656-1.674)	0.839	–	–
Operation				
No	1 (reference)		–	–
Yes	0.991 (0.638-1.538)	0.967	–	–
Treatment lines				
1 line	1 (reference)		1 (reference)	
≥2 lines	2.228 (1.391-3.570)	0.001	1.665 (1.005-2.760)	0.048
PD-1 inhibitors		0.948		
Camrelizumab	1 (reference)		–	–
Sintilimab	1.062 (0.560-2.014)	0.853	–	–
Toripalimab	1.183 (0.370-3.779)	0.777	–	–
Treatment type		0.172		
PD-1+ Chemotherapy	1 (reference)		–	–
PD-1+ Target therapy	1.660 (0.976-2.821)	0.061	–	–
PD-1+ Chemotherapy + Target therapy	1.153 (0.678-1.959)	0.600	–	–
NLR				
≤4.748	1 (reference)		1 (reference)	
>4.748	4.947 (3.081-7.943)	<0.001	2.736 (1.451-5.159)	0.002
dNLR				
≤2.214	1 (reference)		1 (reference)	
>2.214	2.708 (1.723-4.256)	<0.001	1.468 (0.829-2.598)	0.188
MLR				
≤0.309	1 (reference)		1 (reference)	
>0.309	2.058 (1.320-3.209)	0.001	0.852 (0.467-1.555)	0.602
PLR				
≤250.505	1 (reference)		1 (reference)	
>250.505	2.278 (1.428-3.636)	<0.001	0.795 (0.436-1.452)	0.456
SII				
≤887.895	1 (reference)		1 (reference)	
>887.895	3.924 (2.515-6.122)	<0.001	2.068 (1.144-3.739)	0.016

### Risk score model for PFS and OS

**Figure 6:**
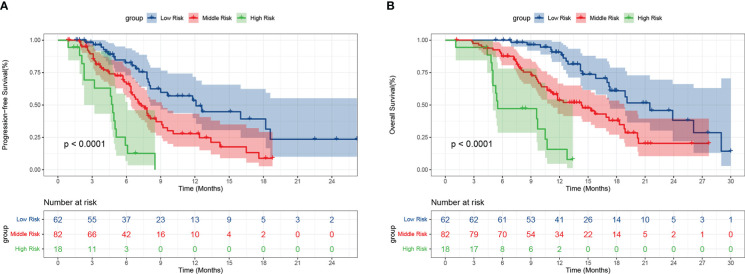
Progression-free survival **(A)** and overall survival **(B)** of patients with different risks. Low risk: score of 0, Middle risk: score of 1 or 2, High risk: score of 3 or 4.

We integrated the variables with P<0.05 into the risk score model for survival according to the multivariate Cox regression analysis. Finally, a total of four risk factors including NLR, ECOG PS, treatment line and lymph node metastasis were included. We assigned a score to the baseline values of the four risk factors mentioned above. A score of 1 was given for each status negatively associated with PFS and OS, namely high NLR, high ECOG PS, second line or posterior, and lymph node metastasis; otherwise, a score of 0 was given. We divided patients into three subgroups based on their scores: low-risk group (score of 0), middle-risk group (score of 1 or 2), and high-risk group (score of 3 or 4). Among the 162 patients, 62 (38.3%) were in the low-risk group, 82 (50.6%) in the middle-risk group, and 18 (11.1%) in the high-risk group. The mPFS was the longest in the low-risk group at 12.1 months (95% CI: 8.009-16.124), followed by 7.3 months (95% CI: 6.201-8.466) for the middle-risk group, and only 4.8 months (95% CI: 4.318-5.349) for the high-risk group (P<0.001) (**Figure 6A**). Regarding OS, the low-risk group had the longest mOS of 21.5 months (95% CI: 16.159-26.907), followed by the middle-risk group with 14.3 months (95% CI: 10.914-17.752) and the high-risk group with only 5.5 months (95% CI: 0.199-10.734) (P<0.001) (**Figure 6B**).

## Discussion

Immunotherapy has been widely applied to cancer treatment, but ideal biomarkers to evaluate its efficacy and predict patients’ outcomes are lacking. Serum inflammatory markers have the advantages of being convenient, repeatable and inexpensive in comparison to current biomarkers like PD-L1 and MSI. Thus, their predictive or prognostic value has been systematically analyzed in a variety of cancers such as NSCLC (26), renal cancer (27), as well as gastric cancer (28). Similar studies were also performed in EC patients treated with immunotherapy. For example, Liu et al. revealed that PLR, NLR, and SII at baseline were significantly linked to PFS and OS (29). Previous researches suggested that pretreatment high NLR was correlated with inferior PFS and OS (30–32). However, another retrospective study indicated that NLR at 6 weeks post-treatment but not at baseline was related to poor PFS (33). Interestingly, Xu et al. suggested that patients with high NLR both at baseline and at 6 weeks post-treatment had a worse PFS and OS compared to those with low NLR (5). To sum up, the prognostic value of these inflammatory biomarkers is still inconsistent and requires further elucidation. Thus, we performed this retrospective study to comprehensively explore the relationship between diverse inflammatory markers and clinical outcomes of immunotherapy for advanced or metastatic ESCC patients.

According to short-term outcomes, our research showed that inflammatory markers such as NLR, dNLR, PLR, and SII levels significantly decreased in PR, ORR, and DCR groups. This finding indicated that inflammatory markers may be related to the short-term outcomes of immunotherapy. Notably, inflammatory marker changes may be influenced by the bone marrow toxicity, which may affect the results. Interestingly, this downward trend was not seen in the PD group. Conversely, the levels of inflammatory markers all showed an upward trend, although they were all not statistically significant. This may be because of the small number of patients in the initial PD group, which may weaken the statistical efficacy. Thus, we further analyzed the changes in inflammatory markers in all PD patients. As shown in **Figure 4**, in contrast to baseline, NLR, dNLR, MLR, and SII were significantly increased when anti-PD-1 therapy failed. These results indicated that the alterations in inflammatory markers might be depended on immunotherapy efficacy and not interfered with bone marrow suppression. In conclusion, the levels of inflammatory markers were significantly decreased when the optimal effect was achieved than at the baseline, however, these values increased again when the disease progressed. The dynamic alterations in inflammatory markers exhibited potentiality in predicting short-term outcomes of immunotherapy and disease progression for patients. To our knowledge, our research is the first study to comprehensively assess dynamic changes and prognostic values in various kinds of inflammatory markers in advanced or metastatic ESCC patients receiving anti-PD-1 treatment. Specifically, we select three-time points including baseline, optimal effect, and when the disease progressed, rather than a fixed time point (5, 33) to assess the changes in inflammatory biomarkers, which seem to be more reasonable and convenient in clinical practice.

As is known, NLR can reflect the systemic inflammatory burdens in patients during cancer development and progression. The prognostic value of NLR for cancer patients has been widely studied. Previous researches revealed that high NLR level has been consistently linked to a worse prognosis in EC when patients were treated with either topical therapies or systemic chemotherapy (34, 35). Our results, consistent with these researches (29, 30), showed that a high baseline NLR was in line with inferior survival in ESCC receiving immunotherapy. Multivariate cox analysis indicated that baseline NLR was an independent prognostic indicator for both PFS and OS. As a novel inflammatory marker based on neutrophils, platelets, and lymphocytes, SII could objectively reflect the balance between inflammation and immunity (36), and thus has an important prognostic value in many types of cancer (37). Our finding confirmed that high SII was a worse prognostic indicator for OS in ESCC patients receiving immunotherapy (29, 38). Notably, based on multivariate Cox regression analysis, both dNLR and PLR, which were positive predictors of short-term outcomes, failed to demonstrate statistical significance for survival. It is potentially attributed that the number of patients is limited and the conversion of short-term outcomes to survival benefits is influenced by many factors.

The clinical features of the patients, such as age, ECOG PS, metastatic sites, treatment line, drug differences and so on, may be also associated with the treatment response and prognosis. Our results indicated that patients with lymph node metastasis negative had significantly better survival than those with lymph node metastasis positive, which supported the previous findings that lymph node metastasis was a worse prognostic indicator in EC patients accepting surgery (39, 40), radiotherapy or radiochemotherapy (41). Notably, previous studies demonstrated that patients with liver metastases were insensitive to immunotherapy in various cancers such as melanoma (42), NSCLC (43), and urothelial carcinoma (44, 45). Additionally, Bilen et al. reported that liver metastasis was linked to poorer OS in advanced stage cancer patients receiving immunotherapy (46). However, there was no significant difference in survival among patients with other metastatic sites, including liver metastases. One possible explanation may be that the sample size in subgroups according to metastatic sites is relatively small, which may lead to statistical insignificance. The correlation needs to be validated in further prospective and large sample size study. The ECOG PS score is a robust indicator of physical status and symptom burden. Patients with lower scores often show a higher tolerance of anti-PD-1 therapy with a better prognosis. Our results revealed that the survival of patients with low ECOG PS score significantly improved after anti-PD-1 therapy, and was therefore considered a significant prognostic indicator for PFS and OS. In accordance to our findings, patients with ECOG PS≥2 had poorer PFS and OS than those with ECOG PS ≤ 1 (30). Regarding treatment lines, we found that the PFS and OS of patients accepting first-line anti-PD-1 treatment were significantly longer than those accepting second-line or posterior treatment. Similarly, multivariate cox regression analysis showed that first-line anti-PD-1 therapy was a significant prognostic indicator for PFS and OS. These findings indicated that advanced or metastatic ESCC patients should receive immunotherapy as early as possible, which may enhance efficacy and thus prolong survival.

Although the prognostic value of inflammatory markers in immunotherapy has been extensively studied. Unfortunately, to date, no prognostic scoring system has been established that can provide multiple information including inflammation, immunity and clinical features. In our study, we develop the first prognostic scoring system based on clinical features and routine blood examination to predict survival outcomes in advanced or metastatic ESCC patients receiving anti-PD-1 treatment. As shown in **Figure 6**, the higher the patient’s risk category, the worse their PFS and OS. This risk model has significant clinical implications for immunotherapy in advanced or metastatic esophageal cancer patients. First, all parameters can be easily performed in clinical practice before treatment, which enriches the prognostic value of inflammatory biomarkers in immunotherapy. What is more, it will provide important survival information for patient classification and may contribute to the identification of patients who will benefit from immunotherapy, and thus may make treatment strategy more reasonable.

Some limitations should be noted in this study. First of all, the patients were enrolled in a single institution, and the sample size was small, so selection biases may have been present. Secondly, levels of various inflammatory markers may be affected by other conditions, thus these confounding factors may have an impact on the conclusions. Lastly, the collection of external information required ethical approval, follow-up, etc. It was difficult for us to perform an independent cohort validation. For confirmation of the conclusions, further multi-centre prospective studies with larger sample sizes are needed.

## Conclusion

In conclusion, the decline in NLR, dNLR, PLR, and SII levels was related to the short-term outcomes of anti-PD-1 treatment, and the elevation of NLR, dNLR, MLR, and SII was indicative of disease progression. The Cox regression analysis indicated that NLR, lymph node metastases, ECOG PS, and treatment lines were significant prognostic indicators for PFS and OS. Based on the above outcomes, we developed a simple and applicable risk score model to evaluate the survival of advanced or metastatic ESCC patients receiving anti-PD-1 treatment, thus enriching the prognostic value of inflammatory biomarkers in immunotherapy.

## Data availability statement

The raw data supporting the conclusions of this article will be made available by the authors, without undue reservation.

## Ethics statement

This study was performed in line with the principles of the Declaration of Helsinki. The studies involving human participants were reviewed and approved by the ethics committee of Anhui Medical University (NO. Quick-PJ 2022-14-35). Written informed consent was obtained from the patients.

## Author contributions

KG and YZ planned and designed the study. LD collected and analyzed the data and wrote the article. ZQ and CZ helped to collect data. ZQ, YS, and WH helped to analyze the data. All authors contributed to the article and approved the submitted version.
